# Demonstration of an app-delivered digital therapeutic program for methamphetamine use disorder

**DOI:** 10.3389/fpsyt.2023.1176641

**Published:** 2023-07-13

**Authors:** Kristin Muhlner, Jeff DeFlavio, Alfonso Ang, Michael Zito, A. Thomas McLellan, Brian Perrochet

**Affiliations:** ^1^Affect Therapeutics, Inc., New York, NY, United States; ^2^UCLA Department of Psychiatry and Biobehavioral Sciences, Los Angeles, CA, United States; ^3^Treatment Research Institute, Research Park, NC, United States

**Keywords:** methamphetamine, digital health, app, craving, outcomes, contingency management, substance use disorder

## Abstract

This study assessed the feasibility and utility of a digital, all-virtual program designed for treatment of methamphetamine use disorder (MUD). Forty-nine adults with moderate- to severe-level MUD (per DSM-5 criteria) commenced the 8-week intervention. All aspects of the program were delivered via smartphone-based app. Intervention components included counseling (cognitive behavioral therapy in group and individual sessions), app-based therapeutic tasks, remote biological drug testing, medical oversight by psychiatrists/nurse practitioners, and contingency management procedures (including rewards for methamphetamine-free saliva drug tests, accomplishing tasks, and engaging in assigned activities). Of the 49 participants who commenced treatment, 27 participants (55%) completed the program. Repeated-measures mixed-model analyses show that participants were more likely to test negative for meth use from week 1 to week 8 (OR = 1.57, 95% CI [1.28, 1.97]; *p* = 0.034). Well-being and social functioning improved among the majority of participants. These results demonstrate the utility of the all-virtual, digital therapeutic program and its ability to help individuals with MUD to reduce or cease methamphetamine use. The program was efficiently implemented and was well received by participants and clinical personnel, indicating its ability to deliver comprehensive, effective care and to retain the difficult-to-engage population of persons with MUD. Of the 27 completers, 16 responded to a 1-month follow-up survey and reported no meth use in the month since completing the program.

## 1. Introduction

Use of methamphetamine and other illicit stimulants is a major contributor to the extent and severity of the nationwide impacts of substance use disorders (SUDs). Methamphetamine (meth) is increasingly implicated in drug overdose mortality and morbidity across the United States (1), and meth has become prevalent among polydrug users (2, 3). Conservative estimates indicate that more than 1.6 million meth users in the nation are in need of treatment, but fewer than 32% of them receive any formal treatment (4, 5).

Effective treatment for individuals with stimulant use disorder remains an elusive target, and no medication has been approved by the FDA for the treatment of patients with methamphetamine use disorder (MUD). Clinicians rely on behavioral therapies that have only limited effectiveness among the diverse populations of stimulant users, especially for those with MUD. Cognitive behavioral therapy (6), contingency management [CM; (7, 8)], individual/group counseling/motivational interviewing (9, 10), community reinforcement approaches [CRA; e.g., (11)], and mutual-support groups (12) are the predominant treatment modalities. Most MUD treatment programs include combinations of some or all of these components in different permutations, many based on the Matrix Model (13, 14). Still, no single modality or combination has been consistently effective in retaining individuals with MUD in treatment, partly because traditional models of treatment delivery require frequent in-person attendance at clinical settings, which is a barrier to engagement and can inhibit retention, compromising outcomes.

Retention in care is a particular challenge for MUD treatment programs. A meta-analysis of outcomes across modalities and SUDs from 1965 through 2016 found that an average of 30% of patients did not complete treatment, but meth users dropped out sooner and completed at lower rates than patients with other SUDs (15–17). Recent research using data from the 2019 Treatment Episode Data Set (TEDS) found that only 31.2% of individuals with stimulant use disorder (excluding cocaine) completed traditional outpatient programs (18). Meth use negatively affects retention among patients in treatment for other SUDs (19, 20).

Improvements are necessary in the design and delivery of behavioral therapies that have proven effective for MUD (21). Toward that end, Affect Therapeutics developed and tested a comprehensive, patient-centered, therapeutic program designed specifically for treatment of individuals with MUD. Services are delivered via a HIPAA-compliant app and include several key therapeutic components (CBT, CM, CRA, and group/individual counseling). Digital healthcare using internet-connected devices enables innovative delivery of SUD treatments (22, 23).

Several digital healthcare platforms have shown promise in delivering therapeutic components and in helping patients stay involved in treatment (24, 25), including patients with MUD (26, 27). Among the most broadly applied digital technologies are mobile health applications (apps), which typically operate on a smartphone platform that is ubiquitous, inexpensive, adaptable, and user friendly (28). Apps can efficiently deliver treatment services, including CM (7). CM has been shown to be the most useful modality for retaining patients in outpatient treatment for MUD (29, 30).

This article reports results of a field study that implemented the Affect digital therapeutic program to assess its feasibility and utility for treatment of MUD.

## 2. Materials and methods

### 2.1. Study design

This single-group demonstration study assessed the feasibility and clinical utility of a digital therapeutic program (developed by Affect Therapeutics, Inc.) delivered by HIPAA-compliant smartphone-based app. The app-based program (see details in “Procedures” Intervention below) includes behavioral components—CM, CBT, CRA, group/individual counseling—that are employed in usual care for MUD. Reduction in meth use and cessation of meth use were measured by self-report and remote, video-supervised saliva drug tests twice weekly during the 8-week program. The study protocol was approved by the WCG Institutional Review Board. From the beginning of screening to the end of the intervention period, the study took 6 months.

Primary outcomes were: *Retention–*measured as program completion—was the primary outcome for establishing the utility and feasibility of the Affect program. *Participation**–***involvement in protocol-specified program activities was measured by participant encounter attendance and interaction with the app during the 8-week intervention (as an additional indicator of feasibility). *Effectiveness*–preliminary demonstration of effectiveness in terms of reduction or cessation of meth use was measured as intra-participant change in meth use, including duration of days abstinent from meth.

### 2.2. Participants

**Figure 1:**
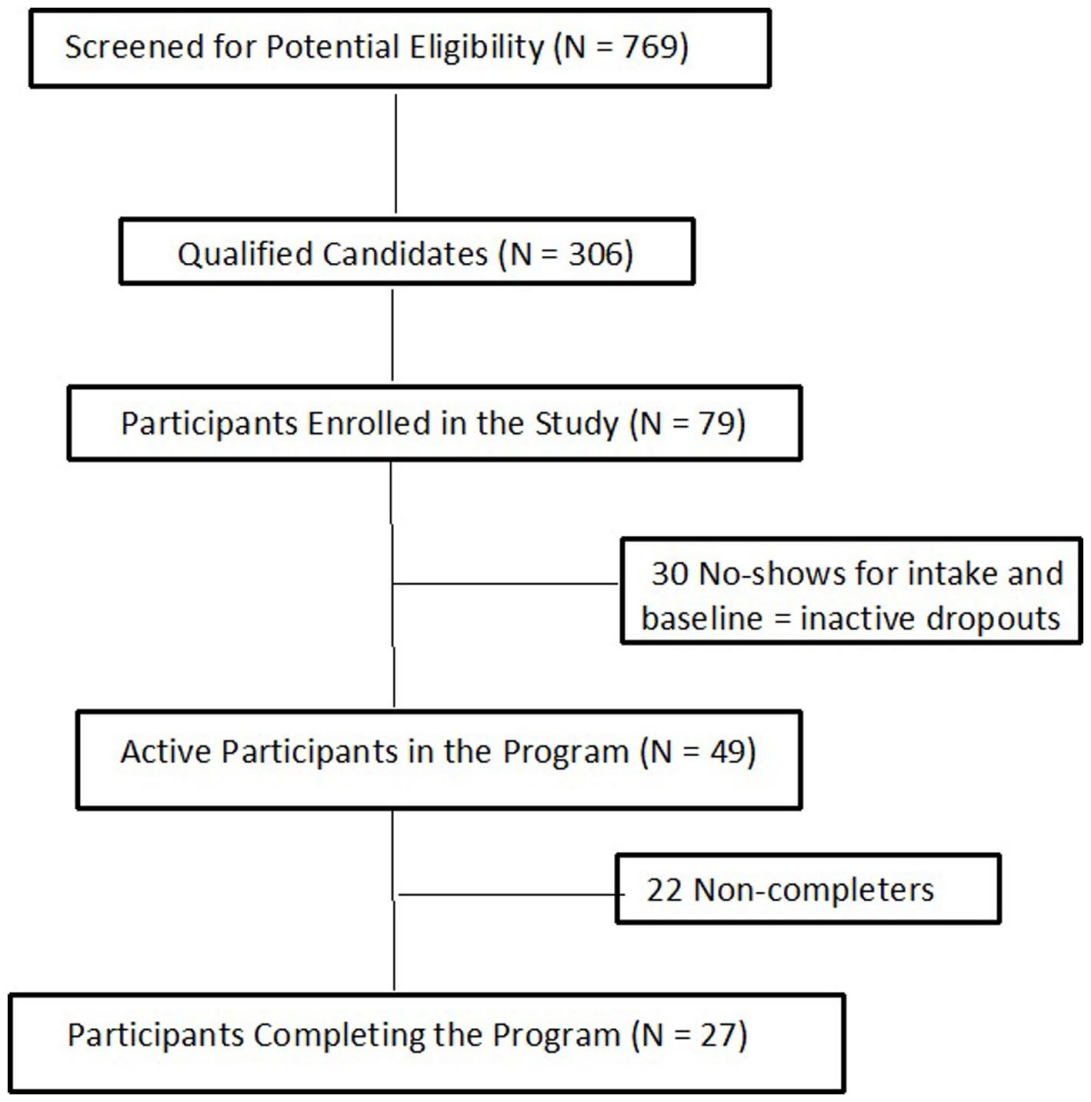
Recruitment flow diagram–participant Progression22 non-completers.

Participants were recruited through multi-media advertising and by word of mouth. Study participant flow is presented in Figure 1. Of the 306 qualified respondents who were remotely screened and assessed for final eligibility, 79 submitted electronically signed consent forms and enrolled. On the intake day required for program involvement, however, 30 individuals did not “attend” the scheduled intake appointment, including assessments and program orientation. Based on the study requirement for enrolled individuals to complete the intake, the 30 who failed to engage were dropped from the study per criteria; thus, the final sample consisted of 49 participants (see Figure 1). Inclusion criteria were: age 18 years or older, able to read and understand English, be an active meth user who met DSM-5 criteria for moderate- to severe-level MUD, affirm an intent to cease meth use, have a smartphone and be capable of using apps, have a mailing address, have medical insurance coverage, and reside in California. Exclusion criteria were: presence of any serious medical or mental health conditions, co-occurring opioid use disorder (moderate to severe level), and involved in any other SUD treatment program. Detailed Inclusion/exclusion criteria appear in Supplemental material/Appendix.

### 2.3. Procedures

Upon completion of screening and consents, participants were enrolled, were provided the Affect app and orientation (guidance on use of the app and brief refresher on program parameters covered in informed consent), and assigned to a licensed counselor and to a medical provider. Participants were asked to use the app to attend twice-weekly group therapy, once-weekly individual therapy, and one visit (remote) with the medical provider to evaluate overall health and ensure the study did not impose risk to the individual. Additionally, all participants were mailed saliva drug test kits [Medical Disposables (31)] to be self-administered under the supervision of the counselor during the individual therapy sessions each week.

All appointments were managed and accessed via the Affect app and conducted over secure video conference. Individual sessions were 30 min and group counseling sessions were 1 h, all occurring synchronous (live), accessed via the app, and conducted by a licensed addiction counselor. Topics followed a general curriculum derived from the Matrix Model (32). At the conclusion of each appointment, the counselor or medical provider marked attendance in the Affect system and the results of the saliva drug test, if completed. For each appointment marked as attended, the Affect system delivered a small financial reward to the member, who was notified via the app immediately upon delivery. Rewards were redeemable to the participant’s CashApp account. For all negative drug screens, the Affect system calculated the appropriate financial incentive and automatically delivered the reward to the participant via the Affect app. Consecutive streaks of negative drug screens were rewarded with a bonus incentive.

### 2.4. Intervention: the Affect digital therapeutic program for MUD

The 8-week Affect program of care for MUD was developed by board-certified addiction psychiatrists and clinicians with extensive expertise in treating patients with MUD and other SUDs. The Affect app delivers all program components, including CM to engage and retain patients while eliciting behavioral change through incentives provided for stimulant-negative results of drug tests and for participation in program activities (e.g., counseling). Participants received app-based prompts for a daily series of behavioral therapeutic tasks and patients engaged with the app and did the incentivized tasks at times convenient to them.

Each day, the Affect app notified the participant of 3–5 new tasks to be completed. These tasks are part of a broader curriculum of content designed to support the participant’s recovery and enable Affect to gather critical participant data necessary to inform care. For example, data collection tasks include basic surveys to gather data regarding daily cravings, substance use, and emotional state. Education tasks include verbal and video content related to the stages of recovery, including topics such as withdrawal management. Motivational tasks include brief verbal or video content coupled with reflection questions, such as reflections on triggers. Skill-building tasks include challenges to promote new, healthy behaviors, such as regular sleep, exercise, and community support group participation.

Program tasks were designed to support behavior change regarding drug avoidance, relapse prevention, prosocial activities, and other lifestyle behaviors that inhibit drug use and enhance life without drugs. Tasks were monitored via the app for satisfactory completion. The timely completion of a task was rewarded at $0.50 each. Behavioral therapy tasks were monitored by the app and by the Care Team for satisfactory completion and verified via direct review of responses in the app (e.g., survey responses). The maximum possible compensation for tasks was $140. Saliva tests were incentivized at a scaled rate (increasing with consecutive tests negative for meth from $10–$30) with $340 maximum possible compensation. Of a total possible compensation of $480 during the intervention period, the average “earned” was <$250.

The app provided a framework to help structure participants’ time in treatment and recovery, offering a user-friendly calendar of upcoming appointments for therapeutic sessions such as group counseling sessions or appointments with psychiatrists and other healthcare providers. Participants engaged in weekly telemedicine sessions with clinical personnel for individual and group therapy.

### 2.5. Measures

#### 2.5.1. Meth use

Meth use was measured by self-report (at intake into the study and twice weekly during the intervention) and by remotely conducted, twice-weekly drug tests during the intervention period using 5-panel saliva-based drug tests [Medical Disposables (31)].

#### 2.5.2. Retention

Retention was a binary measure of completion at the end of the 8-week intervention. Participants who withdrew and those who were terminated for unexplained non-response to Affect personnel (or inaction per app-directed activities) for more than seven consecutive days were deemed non-completers.

#### 2.5.3. Craving for meth

The Visual Analog Scale (VAS) was used to measure craving for meth (33). Patients were asked to mark their response on a line from “0” for “not at all” to “10” for “extremely high” in response to the following question, “How much do you feel the urge to use meth?” Self-reported craving for meth was measured at baseline (intake), and at least once weekly.

#### 2.5.4. Baseline covariates

Baseline covariates were collected at study intake. These covariates were used to control the outcomes used in the analyses. The covariates included sociodemographic factors (age, gender, ethnicity/race, employment, sexual orientation, and family structure such as being a parent or currently being involved in a relationship), and substance use severity factors (baseline meth use and meth craving). The sociodemographic and substance use severity factors were collected as part of a battery of baseline measures administered to participants after study enrollment; all participants self-reported current meth use at intake.

### 2.6. Statistical analyses

Statistical analyses were conducted using SAS 9.4 and STATA 16 software. For all statistical analyses, statistical significance alpha was set at 0.05 using two-tailed hypothesis-testing analyses.

#### 2.6.1. Examination of participant characteristics

Statistical diagnostics were performed on participant characteristics, including sociodemographic variables. Normality assumptions for continuous variables were examined and tested using the Shapiro–Wilk test for normality. *T*-tests were used to test for differences in participant characteristics for the continuous variables, which were age and craving score. Chi-square tests were used to test for differences in the categorical variables of gender (males vs. females) and employment (employed vs. not employed), race/ethnicity, sexual orientation, being a parent (yes vs. no), and in a relationship (yes vs. no). Results showed no statistically significant differences in characteristics among participants who finished the 8-week program (*N* = 27), those who did not complete the program (*N* = 22), or those who did not “attend” the required intake appointment meeting and were dropped from the study (*N* = 30; see Table 1).

**Table 1:** Demographic characteristics of eligible/enrolled participants (*N* = 79).

Characteristic	Completers (*n* = 27)	Non-completers (*n* = 22)	No-Shows in Day 1/Week 1^a^ (*n* = 30)	Test Statistic^b^	*p*-value
*Age,* M (SD)	44.4 (9.8)	37.9 (7.9)	41.6 (12.2)	*F*(78) = 1.79	0.17
*Gender, N (%)*					
Male	10 (37.0%)	10 (45.5%)	14 (46.7%)	*Χ*^2^(4) = 1.75	0.78
Female	14 (51.9%)	10 (45.5%)	11 (36.7%)		
Not Reported	3 (11.1%)	2 (9.0%)	5 (16.6%)		
*Race/Ethnicity, N (%)*					
White	12 (44.5%)	7 (31.8%)	8 (26.7%)	*Χ*^2^(4) = 2.59	0.63
Latinx/Hispanic	5 (18.5%)	6 (27.3%)	10 (33.3%)		
Other race	10 (37.0%)	9 (40.9%)	12 (40.0%)		
*Employment*, N (%)					
Employed	9 (33.3%)	8 (36.4%)	8 (26.7%)	*Χ*^2^(4) = 1.38	0.85
Not employed	13 (48.2%)	8 (36.4%)	14 (46.6%)		
Not reported	5 (18.5%)	6 (27.2%)	8 (26.7%)		
*In Relationship, N (%)*					
Yes	12 (44.4%)	13 (59.1%)	11 (36.7%)	*Χ*^2^(2) = 2.59	0.27
No	15 (55.6%)	9 (40.9%)	19 (63.3%)		
*Parent, N (%)*					
Yes	18 (66.7%)	14 (63.6%)	15 (50.0%)	*Χ*^2^(4) = 2.34	0.67
No	8 (29.6%)	6 (27.3%)	12 (40.0%)		
Not reported	1 (3.7%)	2 (9.1%)	3 (10.0%)		
*Sex orientation, N (%)*					
Heterosexual	16 (59.3%)	15 (68.2%)	18 (60.0%)	*Χ*^2^(4) = 2.63	0.62
Gay/Bisexual	10 (37.0%)	5 (22.7%)	8 (26.7%)		
Not Reported	1 (3.7%)	2 (9.1%)	4 (13.3%)		

a Based on the study requirement for enrolled individuals to complete the study intake to participate in the program, the 30 who failed to engage were deemed dropouts; the analysis sample = 49 participants.

b The numbers within the parenthesis in this column denotes the degrees of freedom.

#### 2.6.2. Comparison of outcomes between completers and non-completers

Comparisons between the 27 completers and 22 non-completers included: number of days in the program, average number of meth-negative drug screens, number of meetings attended, as well as the longest duration of abstinence from meth (consecutive days of no meth use). Normality assumptions for these continuous variables were examined and tested using the Shapiro–Wilk test for normality. Since the distribution of these outcomes was normally distributed, t-test comparisons were made between completers and non-completers.

#### 2.6.3. Meth use outcome differences

Meth use was examined in two ways using results from the saliva tests and from self-report. Logistic mixed models analyses were conducted to examine the repeated assessments of meth use using self-reported meth use at study intake (baseline) and twice weekly during the intervention, and by twice-weekly saliva tests from week 1 to week 8. The meth drug test and the self-reports were measured dichotomously (0 = negative meth use, 1 = positive meth use). Next, logistic repeated-measures mixed models were used to examine meth use outcomes (separately for results of twice-weekly saliva tests during the intervention period and of twice-weekly self-reported meth use) at week 1 to week 8 in comparison to meth use at baseline, controlling for age, gender, race/ethnicity, and employment status, in a relationship, being a parent, and sexual orientation. Finally, in order to assess the quality of the statistical models, the Akaike Information Criterion (AIC), Bayesian Information Criterion, and log-likelihoods were examined. The lower the value of the AIC and BIC, the better the model fits the data. We obtained the AIC and BIC for the null model (or sometimes called the unconditional model, which is the model with just the repeated measures and no covariates) and compared this to the final model.

#### 2.6.4. Craving for meth use

Craving for meth was measured weekly from week 1 to week 8. Linear mixed models were used to assess the repeated measures across 8 weeks controlling for baseline, age, gender, race/ethnicity and employment status, in a relationship, being a parent, and sexual orientation. The craving scores were normally distributed based on the distribution plot and the Shapiro–Wilk test for normality. The quality of the statistical models was also assessed using the Akaike Information Criterion (AIC), Bayesian Information Criterion, and log-likelihood.

## 3. Results

### 3.1. Participant characteristics of completers, non-completers, and non-engaged

Participants who completed the program had a mean age of 44.4 (SD = 9.8) compared with non-completers (mean age = 37.9, SD = 7.9); the dropped individuals who did not engage in the required intake session had a mean age of 41.6 (SD = 12.2). No significant differences were found in terms of age among these three groups (*p* = 0.09). Almost half of participants were females: 51.9% for completers, 45.5% for non-completers, and 36.7% for the individuals who were dropped due to non-attendance at intake. As shown in Table 1, no significant differences were found in terms of gender (*p* = 0.78), race/ethnicity (*p* = 0.86), employment status (*p* = 0.85), being in a relationship (*p* = 0.27), being a parent (*p* = 0.67), or sexual orientation (*p* = 0.75).

### 3.2. Outcomes among completers and non-completers during the 8-week intervention

As a preliminary analysis, we examined differences in outcomes between program completers (*n* = 27) and non-completers (*n* = 22). The average number of days in the 8-week (56-day) program was 55.1 days (SD = 1.61) for completers compared with 21.9 days for non-completers (*t* = 12.3, *p* < 0.001). Significant and more favorable differences were also found between completers vs. non-completers in terms of: (1) the average number of negative drug screens with mean = 5.19, SD = 5.9 for completers vs. a mean = 0.33, SD = 0.79 for non-completers, (*t* = 3.73, *p* = 0.003), (2) the average number of meetings attended by completers (mean = 35.3, SD = 11.6) vs. non-completers (mean = 6.86, SD = 3.73), (*t* = 11.01, *p* < 0.001), and (3) the longest duration of no meth use (consecutive days) for completers (mean = 10.9 days, SD = 8.4) vs. non-completers (mean = 4.5 days, SD = 1.73), =3.51, *p* = 0.001 (see Table 2). As shown in Table 2, no significant differences were found in terms of age (*p* = 0.08), gender (*p* = 0.72), race/ethnicity (*p* = 0.54), employment status (*p* = 0.76), in relationship (*p* = 0.19), being a parent (*p* = 0.70), or sexual orientation (*p* = 0.51).

**Table 2:** Comparison of outcomes between participants who completed the program vs. participants who did not complete the program (*N* = 49).

Outcome measure	Completers (*n* = 27)	Non-completers (*n* = 22)	Test Statistic^b^	*p*-value^a^
Number of days in the Program, M (SD)	55.1 (1.61)	21.9 (13.9)	*t*(47) = 12.3	<0 0.001
Average number of meth-negative drug screens, M (SD)	5.19 (5.90)	0.33 (0.79)	*t*(47) =3.73	0.003
Average number of meetings attended, M (SD)	35.3 (11.6)	6.86 (3.73)	*t*(47) =11.01	<0 0.001
Longest duration of abstinence (number of days of no meth use), M (SD)	10.9 (8.4)	4.5 (1.73)	*t*(47) = 3.51	0.001
Age, M (SD)	44.4 (10.8)	37.9 (12.9)	*t*(47) = 1.92	0.06
Gender, N (%) Male Female Not Reported	10 (37.0%) 14 (51.9%) 3 (11.1%)	10 (45.5%) 10 (45.5%) 2 (9.0%)	*Χ*^2^ (2) = 0.36	0.84
Race/Ethnicity, N (%) White Latinx/Hispanic Other race/ethnicity	12 (44.5%) 5 (18.5%) 10 (37.0%)	7 (31.8%) 6 (27.3%) 9 (40.9%)	*Χ*^2^(2) = 0.96	0.62
Employment, N (%) Employed Not employed Not reported	9 (33.3%) 13 (48.2%) 5 (18.5%)	8 (36.4%) 8 (36.4%) 6 (27.2%)	*Χ*^2^(2) = 0.84	0.66
In Relationship, N (%) Yes No	12 (44.4%) 15 (55.6%)	13 (59.1%) 9 (40.9%)	*Χ*^2^(1) = 1.04	0.31
Parent, N (%) Yes No Not reported	18 (66.7%) 8 (29.6%) 1 (3.7%)	14 (63.6%) 6 (27.3%) 2 (9.1%)	*Χ*^2^ (2) = 0.61	0.74
*Sex Orientatio*n, N (%) Heterosexual Gay/Bisexual Not reported	16 (59.3%) 10 (37.0%) 1 (3.7%)	15 (68.2%) 5 (22.7%) 2 (9.1%)	*Χ*^2^(2) = 1.54	0.46

a Significant at *p* < 0.05.

b The numbers within the parenthesis in this column denotes the degrees of freedom.

### 3.3. Meth use differences (per results of drug tests) between week 1 to week 8 in comparison to baseline reference

**Figure 2:**
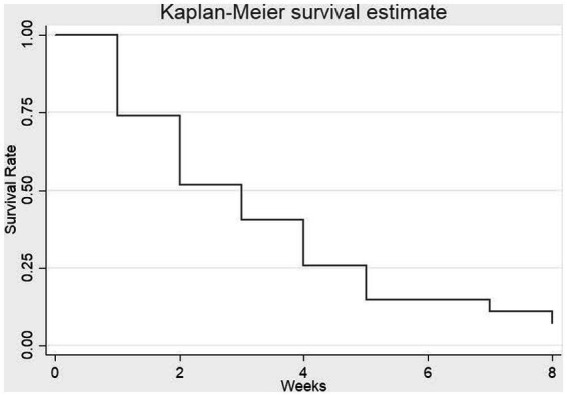
Kaplan-Meir survival plot of % meth positive saliva screens by weeks among completers.

Preliminary analyses were performed to examine the percentage differences in meth-negative test results among participants at baseline reference (i.e., initial saliva test), week 1 to week 8. The meth use test results (binary outcome, where 1 = negative for meth use, 0 = positive for meth use) were examined using logistic repeated-measures mixed-model analyses, where we controlled for age, gender, race/ethnicity and employment, in a relationship, being a parent, and sexual orientation. Results indicate that participants were more likely to test negative for meth use at week 1 to week 8 (Odds ratio = 1.57, 95% CI [1.25, 1.97]; *p* = 0.049) relative to the baseline reference (see Table 3). No significant results were found for age, gender, employment status, in a relationship, being a parent and sexual orientation. A Kaplan-Meir survival plot of the meth-positive saliva screens by week among completers is shown in Figure 2. In the survival plot, the failure parameter is the first positive occurrence for meth after starting in the program.

**Table 3:** Repeated measures meth-negative drug test results (via twice-weekly saliva tests, *N* = 49)^a^.

Assessment variable	Odds ratio	95% confidence interval	*p*-value
*Baseline (Reference* ^*b*^ *)*			
Repeated measures Week 1 to week 8	1.57	[1.25, 1.97]	0.03^c^
Age	1.02	[0.89, 1.18]	0.75
Male	0.98	[0.13, 1.76]	0.23
*Race/ethnicity: white (reference group)*			
Latinx/Hispanic	1.87	[0.42, 10.89]	0.58
Other race	0.24	[0.21, 2.65]	0.24
Employed	2.01	[0.31, 9.96]	0.47
In relationship	0.18	[0.06, 5.14]	0.32
Parent	0.28	[0.03, 7.43]	0.58
Gay/Bisexual	0.35	[0.18, 6.83]	0.49
Log Likelihood	−46.8		
AIC (Null model) AIC (Full model)	155.2 115.6		
BIC (Null model) BIC (Full model)	160.8 145.5		

a Includes the analysis sample of 49 who attended at least the first week of the program.

b Reference for baseline is the first saliva drug test during the intervention period.

c Significant at *p* < 0.05.

### 3.4. Differences in self-reported abstinence from meth (no meth use) between week 1 to week 8, in comparison to baseline self-reported abstinence from meth

Preliminary analyses were performed to examine the percentage differences in self-reported negative meth use among participants at baseline (at intake, preceding intervention period), week 1 to week 8. Self-reported meth use (binary outcome, where 1 = negative for meth use, 0 = positive meth use) was analyzed using logistic repeated-measures mixed models, where we controlled for age, gender, race/ethnicity and employment, in relationship, being a parent and sexual orientation.

Results indicate that participants were increasingly likely to report no meth use from week 1 to week 8 (Odds ratio = 1.69, 95% CI [1.29, 2.09]; *p* = 0.024) relative to the baseline reference (at intake) (see Table 4). No significant results were found for age, gender, and employment status, in a relationship, being a parent and sexual orientation.

**Table 4:** Self-reported abstinence from meth (no meth use) (*N* = 49).

Assessment period	Odds ratio	95% confidence interval	*p*-value
*Baseline (Reference)* *^a^*			
Repeated measures Week 1 to week 8	1.69	[1.29, 2.09]	0.031^b^
Age	0.98	[0.89, 1.09]	0.68
Male	0.29	[0.07, 1.27]	0.11
Race/ethnicity: white (reference group)			
Latinx/Hispanic	1.89	[0.41, 10.74]	0.51
Other race	0.46	[0.11, 2.12]	0.32
Employed	1.89	[0.56, 6.11]	0.30
In relationship	0.12	[0.03, 5.89]	0.36
Parent	0.26	[0.02, 7.28]	0.56
Gay/Bisexual	0.31	[0.15, 6.97]	0.52
Log Likelihood	−59.9		
AIC (Null model) AIC (Full model)	155.2 115.6		
BIC (Null model) BIC (Full model)	160.8 145.5		

a Baseline is self-report of no meth use at study intake (preceding the intervention period).

b Significant at *p* < 0.05.

### 3.5. Self-reported average craving scores from week 1 to week 8 in comparison to baseline craving

Preliminary analyses were performed to examine self-reported craving ratings among participants at baseline, week 1 to week 8. At baseline, participants reported an average craving score of 3.69, SD = 1.89. Using linear mixed models repeated measures analyses, results show that the change in craving scores was not significantly different between week 1 to week 8 (*t* = 1.732, 95% CI [−1.78, 5.24]; *p* = 0.26) compared to the baseline craving score. No significant results were found for age, gender, and employment status, in a relationship, being a parent and sexual orientation. See Table 5.

**Table 5:** Average craving score during the assessment period (*N* = 49).

Assessment periods	Mean (SE)	95% confidence interval	*T*-statistic	*p*-value
*Baseline (reference)* *^a^*				
Repeated measures Week 1 to Week 8	1.73 (1.79)	[−1.78, 5.24]	1.12	0.26
Age	0.04 (0.23)	(−0.41, 0.49)	1.79	0.07
Male	−0.14 (0.39)	[−0.90, 0.62]	−0.35	0.73
*Race/ethnicity: white (reference group)*				
Latinx/Hispanic	−0.15 (0.41)	[−0.95, 0.65]	−0.38	0.70
Other race	−0.18 (0.51)	[−1.18, 0.82]	−0.36	0.72
Employed	−0.75 (0.93)	[−2.57, 1.07]	0.72	0.48
In relationship	−0.67 (0.41)	[−0.13, 1.47]	−1.65	0.10
Parent	−0.42 (0.57)	[−1.54, 0.69]	−0.75	0.45
Gay/Bisexual	0.33 (0.49)	[−0.63, 1.29]	0.68	0.50
				
Log likelihood	−638.9			
AIC (Null model) AIC (Full model)	1896.4 1792.1			
BIC (Null model) BIC (Full model)	1902.3 1835.5			

a Baseline craving reported at study intake (preceding the intervention period).

## 4. Discussion

This demonstration study occurred during the first 6 months of 2021 when traditional in-clinic SUD treatment was still constrained by COVID pandemic restrictions and limited vaccination. The all-virtual nature of the program eliminated the requirement for patients to frequently appear in person in a clinic setting, which can inhibit initial engagement of individuals with MUD and also imposes a burden that can reduce long-term retention in treatment. That flexibility may have been a factor in the intense interest in the study; 769 persons responded to study announcements, and 306 were screened as potentially eligible candidates. Beyond the planned 48-person sample, we enrolled a total of 79 eligible individuals, all with moderate/severe-level MUD. Although 30 did not follow through with intake and thus never commenced the intervention, there were no significant differences between them and the 49 in the analysis sample. Below we discuss the results in context of the main outcomes.

### 4.1. Retention

Consistent with accepted approaches to measure retention in SUD treatment [e.g., (34, 35)], the outcome was based on *completion* as a binary measure at the end of the 8-week intervention. Termination or unexplained non-response to Affect personnel (or app-directed activities) for more than seven consecutive days in any period of the 8-week study period equated to “non-completer” status for the purpose of determining retention. The Lappan study (15) reported that the average dropout rate among patients with MUD in all forms of treatment from 1965 to 2016 was 53.5%, inferring a 46.5% completion rate. More recent data from the 2019 Treatment Episode Data Set [TEDS; SAMHSA (18)] show that only 31.2% of individuals were retained in (completed) outpatient programs for stimulant use disorder (other than cocaine), indicating a nearly 68.8% dropout rate. The Affect program completion rate was 55.1%, comparing favorably to 31.2% completion of outpatient programs for MUD shown in the 2019 TEDS data, indicating the Affect dropout rate of 44.9% versus the 68.8% rate in TEDS. The CM component is a strong factor in retention, which is consistent with findings of other literature on MUD programs [e.g., (29, 36–38)]. Our results confirm the feasibility of the Affect program’s remote management of CM procedures, which were conducted via app, including the delivery of rewards for completion of tasks and activities.

### 4.2. Participation

In addition to retention (treatment completion vs. dropout/withdrawal), *participation* was a construct composed of the number of “attended” therapeutic tasks/activities per the Affect app relative to not-attended events such as: scheduled/planned virtual interactions for program activities (e.g., counseling, group meetings), completed remote drug tests, and kept appointments with referred service providers (e.g., psychiatrists, physicians). Participants who consistently engaged in program activities were more likely to complete the program and reduce meth use according to self-report and drug tests; the 27 completers attended an average of 35 meetings compared to less than 7 attended by non-completers. Overall, more than 41% of all activities and tasks were attended, 8% were not attended due to cause or cancelation, and more than 49% were not attended.

### 4.3. Effectiveness

The effectiveness measure is a composite of self-reported meth use (daily Yes/No) and meth-negative saliva test results obtained twice weekly over the course of the program. A participant who provided at least four consecutive meth-negative saliva tests during the final 4 weeks of the 8-week program met criteria for positive reduction in meth use at a significant level, indicating within-participant effectiveness. Reductions in saliva test meth use across the analysis sample were statistically significant. These findings were consistent with self-reported meth abstinence with a Kappa test of concordance statistic of 0.78.

### 4.4. Meth craving

Analyses could not detect significant differences in craving trajectories, but subgroups appeared to have erratic patterns in which early-phase high craving scores were *not* associated with relapse to use of meth nor with dropout. Notably, participants with higher craving appear to stay in the program longer. The apparent paradox that high craving did not associate with relapse to meth is intriguing but statistically unsubstantiated.

### 4.5. Sustainability

At exit interviews, 12 participants expressed interest in continuing in the Affect program and were offered the opportunity to remain for another 8 weeks but without the CM component. All 12 were retained without CM in the post-study program, which suggests the program’s viability and acceptability to people with MUD. The commercialization of the Affect program for MUD is being explored in a NIDA-funded Small Business Innovation Research project.

#### 4.5.1. Global improvement

General well-being was measured by end-of-treatment administration of the Treatment Effectiveness Assessment (39) using a 1-to-10 scale of responses to questions in four domains related to recovery. Thirty-nine percent of respondents rated their improvement in the drug use domain at a “10” (much better) and 52% rated their improvement in overall health at a “10”.

*Preliminary Results of Follow-ups.* Of the 27 completers, 16 responded to 1-month follow-up surveys; ~56% of respondents self-reported no meth use in the month since program completion.

## 5. Conclusion

The results of the study indicate that in this sample of individuals with MUD, the Affect digital therapeutic program for MUD was feasible to administer, was acceptable to participants, was effective in retaining participants in the program, and helped the majority of program participants to significantly reduce meth use. To thoroughly demonstrate effectiveness of the Affect program for MUD, a larger scale test would compare the Affect program against a research-tested MUD care program [e.g., the Matrix Model; (17)]. Results of this study may be useful to inform the development of and substantiate the practice of dHealth/mHealth-assisted treatment for MUD.

## Data availability statement

The original contributions presented in the study are included in the article/Supplementary material, further inquiries can be directed to the corresponding author.

## Ethics statement

The Study involving human participants was reviewed and approved by Western Institutional Review Board (WCG). The patients/participants provided their written informed consent to participate in this study.

## Author contributions

KM contributed to planning, conceptualizing, and writing the manuscript. JD helped in conceptualizing and reviewing the manuscript. AA performed the data analysis and helped to write the manuscript. MZ provided comments and reviewed the manuscript. AM assisted in conceptualizing and reviewing the manuscript. BP contributed to conceptualizing and writing the manuscript. All authors contributed to the article and approved the submitted version.

## Funding

This study was funded by Affect Therapeutics, Inc.

## Conflict of interest

KM and JD were employed by Affect Therapeutics, Inc.

The remaining authors declare that the research was conducted in the absence of any commercial or financial relationships that could be construed as a potential conflict of interest.

## Publisher’s note

All claims expressed in this article are solely those of the authors and do not necessarily represent those of their affiliated organizations, or those of the publisher, the editors and the reviewers. Any product that may be evaluated in this article, or claim that may be made by its manufacturer, is not guaranteed or endorsed by the publisher.
