# Supplementary material for: Demonstration of an app-delivered digital therapeutic program for methamphetamine use disorder
Source: Front Psychiatry. 2023 Jul 13;14:1176641. doi: 10.3389/fpsyt.2023.1176641 (PMC10373299; doi:10.3389/fpsyt.2023.1176641)
Supplement: Supplementary file 1 [file Data_Sheet_1.pdf]

SUPPLEMENTAL MATERIAL

Inclusion/Exclusion Criteria

Inclusion Criteria:

1. Be 18 years of age or older;
2. Have methamphetamine use disorder (MUD) or documented high-risk methamphetamine use history, confirmed by DSM-5 criteria for stimulant use disorder, methamphetamine type;
3. Have stated interest in reducing and/or stopping methamphetamine use;
4. Have and be able to use a smartphone and agree to download and use the Affect app as part of treatment and study-related procedures;
5. Have a health insurance plan (or Medicaid) to ensure that participants have access to medical care if needed;
6. Be English speaking and have reading capacity sufficient to understand explanations of study procedures and the informed consent to participate;
7. Be a California resident with a mailing address or P.O. Box
8. Be able to freely give informed consent and be willing to electronically sign the digital informed consent to participate in the study;
9. Be willing to comply with study procedures, including use of the Affect app consistent with the study protocol and therapeutic activities.

Exclusion Criteria:

1. Serious medical diagnoses (e.g., seizure, stroke, heart disease);
2. Serious mental illness (e.g., schizophrenia, bipolar, active suicidality);

3. Moderate- to severe-level opioid use disorder or alcohol use disorder;
4. Pregnancy;
5. Have been in a prior study of pharmacological or behavioral treatment for MUD within 6 months of study consent;
6. Be concurrently receiving other behavioral or pharmacological services for treatment of MUD;
7. Have pending legal action or other situation that could inhibit consistent participation in the study or in study activities.
